# Fostering emotional well-being in adolescents: the role of physical activity, emotional intelligence, and interpersonal forgiveness

**DOI:** 10.3389/fpsyg.2024.1408022

**Published:** 2024-05-22

**Authors:** Shicheng Yang, Longjun Jing, Qianqian He, Huilin Wang

**Affiliations:** ^1^School of Physical Education, Hunan University of Science and Technology, Xiangtan, China; ^2^Faculty of Economics, Chulalongkorn University, Bangkok, Thailand; ^3^School of Business, Hunan University of Science and Technology, Xiangtan, China

**Keywords:** physical activity, emotional intelligence, interpersonal forgiveness, positive emotions, adolescents

## Abstract

**Introduction:**

Adolescence is considered a stress-sensitive developmental period, and the escalating and sustained pressure during this phase poses a significant threat to the mental and physical well-being of adolescents. Therefore, enhancing positive emotions in adolescents is crucial. This study aims to investigate the impact of physical activity on the emotional intelligence, interpersonal forgiveness, and positive emotions of adolescents.

**Methods:**

Using a cluster sampling method, data were collected from 500 adolescents in four schools across the Xiangxi Tujia and Miao Autonomous Prefecture of Hunan Province, China. A total of 428 valid questionnaires were collected and analyzed. The study employed AMOS v.23 to construct a structural equation model to validate the hypotheses.

**Results:**

The results indicate that physical activity significantly influences the emotional intelligence, interpersonal forgiveness, and positive emotions of adolescents. Furthermore, emotional intelligence and interpersonal forgiveness mediate the relationship between physical activity and positive emotions.

**Discussion:**

Based on these findings, collaborative efforts from government agencies, schools, and families are essential to provide robust support for adolescents’ participation in physical activity, encouraging more adolescents to actively engage in sports.

## 1 Introduction

Over the years, adolescents have been subjected to sustained pressure from internal and external sources in schools, such as academic stress (Ye et al., 2019; Högberg et al., 2020), interpersonal stress (Fiorilli et al., 2019), daily life hassles (Chiang et al., 2019), and educational competition pressure (Högberg, 2021). These pressures often result in negative emotions like anxiety, depression, anger, sorrow, and fear (Reddy et al., 2018; Pascoe et al., 2020). Moreover, a meta-analysis of 41 studies across 27 countries revealed a global mental disorder prevalence of 13.4%, with anxiety at 65% and depression at 26% (Högberg, 2021). With time, the psychological stress burden continues to escalate. Urgent intervention is required, as failure to address this issue not only impacts adolescent health but also perpetuates a vicious cycle hindering their future social development.

However, numerous studies confirm that fostering positive emotions not only helps alleviate the negativity adolescents experience under stress but also broadens their attention and cognitive abilities (Fredrickson, 2004). It enhances adolescents’ psychological health resilience (Zhang et al., 2022). Scholars argue that adolescents should cultivate positive emotions in their lives to alleviate the oppression brought by daily challenges. Effective solutions for negative emotions and hindrances to mental and physical health not only promote individual and collective well-being but also contribute to overall environmental improvement, elevating individuals to higher mental states (Fredrickson, 1998; Tugade et al., 2004). Furthermore, positive emotions motivate individuals to broaden and build their cognitive, attentional, and behavioral repertoires. Unique positive emotional concepts are particularly likely to provide individuals with more informational value than overall emotions (Tan et al., 2022).

Numerous studies indicate that engaging in physical activity offers various benefits for adolescents (Singh et al., 2023). Firstly, it effectively reduces the probability of adolescent depression and alleviates negative emotions (Zhang et al., 2022). Secondly, it enhances adolescents’ self-efficacy and positive well-being (Norris et al., 1992; Steptoe and Butler, 1996; Zhang et al., 2022). Additionally, engaging in 2–2.5 h of high-intensity exercise per week can improve self-esteem and happiness, contributing to anxiety relief in adolescents (Anderson and Shivakumar, 2013). Importantly, regular physical activity enables adolescents to enjoy a higher quality of life and better positive emotions, enhancing their learning efficiency and academic performance in school (Singh et al., 2012).

However, the intensity of physical activity for many adolescents falls far short of the levels required to promote these benefits (Sallis, 2000). Physical education classes are a crucial component of school education, emphasizing their core role and profound impact on the health of almost all adolescents (Pate et al., 2006; Salmon et al., 2007; Van Sluijs et al., 2007). Due to the increasing pressure of academic studies, adolescents spend a significant amount of time on academic subjects, while the time allocated for physical activity continues to decrease. As a result, their physical activity intensity and time fail to meet ideal standards (Singh et al., 2012). Surveys show that the level of moderate-to-vigorous physical activity in physical education classes for adolescents remains low, falling short of the 50% standard recommended by the Centers for Disease Control and Prevention in the United States and the British Sports Association (Van Sluijs et al., 2021). Therefore, it is imperative to prioritize physical activity for adolescents, urging collaboration among governments, schools, and families to encourage and support increased time spent on physical activity in schools. This not only alleviates academic stress but also fosters the healthy development of positive emotions in adolescents.

While both positive and negative emotions are equally important for studying adolescent health, current research on adolescent positive emotions remains limited (Shen et al., 2018; Li et al., 2022). Most studies on adolescent emotions focus on treating depression, anxiety, and fear, with greater attention given to negative emotions in adolescents (Rodriguez-Ayllon et al., 2019). There is a lack of in-depth research and effective improvement suggestions in academia for enhancing adolescent positive emotions. This study posits that adolescents possessing more positive emotions are driven toward success, leading to success in various life domains. In contrast to previous research, this study primarily focuses on how to better enhance adolescent positive emotions through intervention in physical education classes, enabling adolescents to experience positive interpersonal interactions and improve emotional control. Given the gaps and issues identified in previous studies, this research proposes the following objectives: (1) investigate the factors influencing positive emotions in adolescents; (2) explore the relationship between physical activity, emotional intelligence, interpersonal forgiveness, and positive emotions in adolescents; (3) provide feasible suggestions for enhancing adolescent positive emotions through increased physical activity to the government, schools, and families.

## 2 Literature review and hypothesis development

### 2.1 The concept of variables

#### 2.1.1 Physical activity

The definition of physical activity in the literature can be summarized from two perspectives. From a macro perspective, physical activity refer to various sports competitions, daily exercise, and related sports activities (Jia et al., 2019). From a micro perspective, physical activity is defined as any bodily movement produced by skeletal muscles that results in energy expenditure (Singh et al., 2023). Given that the sample for this study consists of adolescents, the definition of physical activities in this research mainly focuses on the micro level, referring to engagement in physical exercises that promote physical and mental health.

#### 2.1.2 Emotional intelligence

The concept of emotional intelligence was introduced in 1990 by Salovey and Mayer (1990), referring to an individual’s ability to monitor, differentiate, and guide their own and others’ feelings and emotions to facilitate thinking and actions (Kotsou et al., 2019). Emotional intelligence is considered an emotional skill, personality trait, and capability for interacting with others, determining how individuals respond to external needs and stress (Bar-On, 2000). Some scholars, such as Law et al. (2004), view emotional intelligence as the ability to evaluate one’s own and others’ emotions and apply emotional regulation skills. In this study, emotional intelligence is defined based on the perspectives of scholars like Law et al. (2004).

#### 2.1.3 Interpersonal forgiveness

Interpersonal forgiveness has been widely studied in psychology, with McCullough et al. (2007) defining it as an individual’s change in pro-social motives following an offense, including a reduction in motives for revenge and avoidance and an enhancement of benevolent motives. It is an effective approach to resolving interpersonal conflicts, going beyond eliminating negative emotions toward the offender to positively altering attitudes and behaviors toward them (Mccullough et al., 2003). Forgiveness, distinct from reconciliation, involves forgiving an individual and reestablishing a positive relationship with them. It signifies a pro-social change toward the offender, with individuals inclined to forgive often being more agreeable and emotionally stable (Riek and Mania, 2012). This study considers forgiveness as a positive psychological response to interpersonal harm, indicating a reluctance to retaliate against the perpetrator and a positive expectation for future relationships with others.

#### 2.1.4 Positive emotion

In psychological studies, positive emotion is defined as a collection of discrete pleasant emotional states, including happiness, satisfaction, excitement, enthusiasm, and active positive emotional states (Carl et al., 2013). This definition is also considered a functional phenomenon that motivates individuals by mobilizing psychological and physiological resources to achieve anticipated goals, aiding in expanding social resources for enhanced happiness (Fredrickson, 1998; Fredrickson, 2001). In this study, positive emotion refers to an emotional state in adolescents, with positive emotional states promoting creativity, subjective well-being, emotional control, and healthy interpersonal development (Fredrickson, 2004). Positive emotion facilitates harmonious and positive relationships, creating a positive and healthy environment for adolescent physical and mental development.

### 2.2 Hypotheses

#### 2.2.1 Physical activity, emotional intelligence, interpersonal forgiveness, and positive emotion

Existing research indicates that regular participation in physical activity can enhance emotional intelligence, strengthen relationships, improve academic performance, and reduce the risk of mental health problems, consequently promoting positive emotions in adolescents (Chan et al., 2019; Cho, 2020; Skurvydas et al., 2021; Li et al., 2022). Furthermore, participating in physical activity has positive effects on the physical and mental health of adolescents, fostering positive interpersonal relationships and improving emotional intelligence, enabling better control of negative emotions (Acebes-Sánchez et al., 2019). Some studies suggest that emotional intelligence is beneficial for character influences on mental health, promoting positive emotions in adolescents and contributing to healthier interpersonal relationships (Ruvalcaba-Romero et al., 2017). However, McCullough et al. (2000) argue that adolescents with higher emotional intelligence can better forgive others, facilitating constructive handling of negative emotional reactions to others’ wrongdoing. Emotional intelligence plays a crucial role in interpersonal forgiveness, with adolescents who can better identify, absorb, understand, and regulate emotions being more adept at managing emotions, cooperating effectively, possessing superior social skills, and solving interpersonal problems more successfully (Karaoglan Yilmaz et al., 2023). This suggests a stronger ability for perspective-taking and a tendency to forgive others for harmful or transgressive behaviors (Hodgson and Wertheim, 2007). Rey and Extremera (2014) also assert that individuals with higher emotional intelligence are better at reducing retaliatory motives, maintaining positive relationships with offenders, and establishing positive interpersonal relationships. Higher levels of emotional intelligence correlate with higher levels of agreeableness, cooperativeness, and forgiveness tendencies. Individuals with high emotional intelligence can view problems from others’ perspectives, thus promoting tolerance (Rizkalla et al., 2008).

Furthermore, research indicates a significant correlation between emotional intelligence and positive emotion. Emotional intelligence enables reflective regulation of emotions, playing a crucial role in promoting positive emotions (Sánchez-Álvarez et al., 2016). Study by Extremera and Rey (2016) suggest a significant correlation between emotional intelligence and positive emotion. Individuals with high emotional intelligence frequently experience pleasant or positive emotions and rarely experience unpleasant or negative emotions. By regulating emotions, emotional intelligence can foster the occurrence of positive emotions and reduce the frequency of negative emotions. Additionally, research by Ruvalcaba-Romero et al. (2017) indicates that emotional intelligence plays a vital role in social environments. Individuals with high emotional intelligence can effectively control their emotions, possess strong social skills, express positive emotions to themselves and others, and increase positive emotions in life. According to the results of Trigueros et al. (2019), adolescents’ emotional intelligence is positively correlated with positive emotion. Emotional intelligence plays a crucial role in regulating and repairing emotions and increasing emotional efficiency, resulting in greater emotional satisfaction, stronger emotional happiness, and a healthier mindset.

Numerous studies suggest a significant correlation between interpersonal forgiveness and positive emotion (Mccullough et al., 2003; Barcaccia et al., 2020). Forgiveness is seen as not only eliminating negative emotions toward the offender but also enhancing the positive attitude and behavior toward them. It involves re-establishing a positive relationship with the offender, representing a pro-social change (Riek and Mania, 2012). Riek and Mania (2012) suggests a relationship between forgiveness and positive emotional outcomes. Higher levels of forgiveness can help individuals move away from negative emotions associated with unforgiveness, enhancing positive emotions and promoting physical and mental well-being. Forgiveness is related to a decrease in physiological arousal and an increase in positive emotion and psychological well-being (Lawler et al., 2005). Most Chinese studies suggest a significant positive correlation between forgiveness and positive emotion (Chen et al., 2017; Gao et al., 2022). Individuals with forgiveness have higher subjective well-being, greater life satisfaction, more positive emotions, and fewer negative emotions, making forgiveness an effective tool for resolving conflicts in interpersonal relationships. Based on the aforementioned relationships between the variables, this study proposes the following hypotheses:

*Hypothesis 1 (H1)*: Physical activity is positively correlated with emotional intelligence.

*Hypothesis 2 (H2)*: Physical activity is positively correlated with interpersonal forgiveness.

*Hypothesis 3 (H3)*: Emotional intelligence is positively correlated with interpersonal forgiveness.

*Hypothesis 4 (H4)*: Emotional intelligence is positively correlated with positive emotion.

*Hypothesis 5 (H5)*: Interpersonal forgiveness is positively correlated with positive emotion.

#### 2.2.2 The mediating effects

Adolescents with higher levels of positive emotion can foster creativity, subjective well-being, emotional control, and healthy interpersonal development (Fredrickson, 2004), thereby enhancing their learning efficiency and academic performance in school (Singh et al., 2012). Numerous studies indicate that regular participation in physical activity can elevate positive emotions in adolescents, concurrently enhancing their emotional intelligence, strengthening interpersonal relationships, improving academic performance, and reducing the risk of mental health problems (Skurvydas et al., 2021; Li et al., 2022). Engaging in physical activity has a positive impact on the physical and mental well-being of adolescents, not only contributing to the establishment of positive interpersonal relationships but also elevating their emotional intelligence, enabling better control of negative emotions (Acebes-Sánchez et al., 2019). Moreover, adolescents with higher emotional intelligence are better able to forgive others, facilitating constructive handling of negative emotional reactions to others’ wrongdoing (Acebes-Sánchez et al., 2019). Emotional intelligence, beneficial for character influences on mental health, promotes positive emotions in adolescents, thereby contributing to healthier interpersonal relationships (Ruvalcaba-Romero et al., 2017; Guerra-Bustamante et al., 2019).

Rey and Extremera (2014) propose that individuals with higher emotional intelligence can better reduce retaliatory motives, maintain positive relationships with offenders, and establish positive interpersonal relationships. Adolescents with stronger forgiveness abilities are more likely to experience positive emotions. Therefore, this study posits the following mediating hypothesis.

*Hypothesis 6 (H6)*: Emotional intelligence and interpersonal forgiveness mediate the relationship between physical activity and positive emotion.

**Figure 1:**
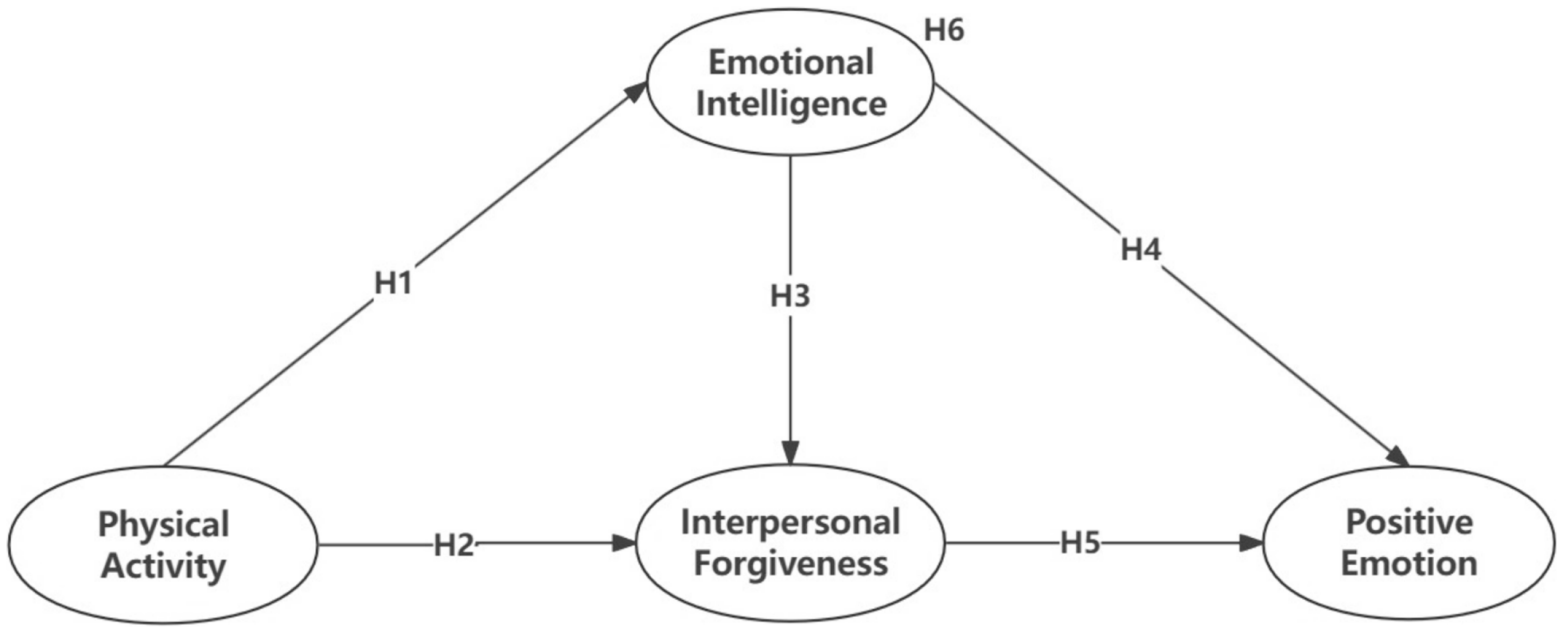
The hypothesized model.

A summary of all hypotheses is presented in Figure 1.

## 3 Methodology

### 3.1 Participants and procedure

The research targeted adolescents aged 12 to 18. From late August to mid-September 2023, the researcher distributed paper questionnaires in four schools in the Xiangxi Tujia and Miao Autonomous Prefecture of Hunan Province, China. All students participated voluntarily, and parental informed consent was obtained before their involvement. As a token of appreciation, each participating student received a notebook upon completing the questionnaire. A total of 500 adolescents from 10 schools were selected for the survey, and 428 valid questionnaires were collected, resulting in an effective response rate of 85.6%.

Table 1 presents demographic information for the 428 participating adolescents, including age range, gender, types of physical activities, and weekly exercise frequency. The demographic statistics in Table 1 reveal that nearly 64.6% of the sample falls within the age range of 16 to 18. Additionally, the survey achieved a balanced representation of both genders. The adolescents’ exercise preferences were predominantly in basketball, soccer, and badminton. Lastly, 48.8% of the adolescents reported not engaging in physical activities on a weekly basis.

**Table 1:** Participant profile (*N* = 428).

Profiles	Survey (%)
Age	
12–15	35.4
16–18	64.6
Gender	
Male	51.8
Female	48.2
Sporting event	
Basketball	28.5
Soccer ball	20.2
Volleyball	14.1
Run	12.3
Badminton	17.1
Others	7.8
The frequency of exercise each week	
0	48.8
1–2	27.5
3–4	14.9
5–7	8.8

### 3.2 Instruments

The questionnaire comprises five sections. The first section gathers information on respondents’ age, gender, types of sports activities, and weekly exercise frequency. The second section utilizes the Physical Activity Scale developed by Sallis et al. (1985), encompassing three items. Sample items include “During the past week, I actively participated in various forms of moderate physical activity, including tasks like sweeping and mopping, as well as engaging in sports such as volleyball, Ping-Pong, and similar activities.” The third section collects respondents’ emotional intelligence data using a scale developed by Davies et al. (2010), consisting of five items. Sample items include “I easily recognize my emotions as I experience them.” The fourth section gathers respondents’ interpersonal forgiveness data using a scale developed by McCullough et al. (1998), comprising four items. Sample items include “I wish that something bad would happen to them; I want to see them hurt and miserable.” Lastly, the fifth section collects data on respondents’ positive emotion, employing a scale developed by Diener et al. (2010), with five items. Sample items include “I lead a purposeful and meaningful life.” All four scales are measured using a five-point Likert scale, with response options ranging from 1 (i.e., strongly disagree, disagree) to 5 (i.e., strongly agree, agree).

To adapt to the Chinese cultural background and context, the researcher made some modifications to certain items in the scales. We removed some items from the scale due to their redundancy and similarity; we only retained items that differed significantly in content. For example, in the scale measuring positive emotions, there were two very similar statements: “I lead a purposeful and meaningful life” and “I am a good person and live a good life.” We ultimately retained “I lead a purposeful and meaningful life” because this item alone effectively captures whether respondents are enjoying and are satisfied with their lives. Reducing repetitive questions was crucial to avoid respondent fatigue and resistance, which can occur when participants perceive questions as overly similar.

To ensure the changes did not compromise the reliability and validity of the questionnaire, a pilot test was conducted prior to the main data collection. This pilot test involved a total of 82 participants, and the results were promising. The Cronbach’s alpha coefficients for the revised scales exceeded 0.8, indicating high internal consistency and confirming the appropriateness of the modifications. This pilot testing was essential not only for verifying the effectiveness of the item reductions but also for ensuring that the revised items were suitably adapted to the cultural context without altering their original intent.

### 3.3 Data analysis

This study employed AMOS v23 to construct a Structural Equation Model (SEM) to examine how adolescents enhance positive emotions through physical activity. Maximum Likelihood (ML) estimation was used to estimate the model parameters. Prior to conducting the ML estimation, the normality of data was assessed to validate the assumption of normal distribution, which is essential for the accuracy of ML estimation. Skewness and kurtosis values were examined for all variables involved in the study. According to commonly accepted guidelines, the absolute value of skewness should be less than 3, and the absolute value of kurtosis should be less than 7 to consider the distribution as normal (Kline, 2005). In this study, the absolute values of skewness ranged from 0.082 to 1.168, and the absolute values of kurtosis ranged from 0.047 to 1.202, all of which are well within the recommended thresholds. Therefore, it can be concluded that the data conforms to a normal distribution, ensuring that these values were within acceptable ranges for assuming normality in the subsequent analyses.

The two-step modeling approach was adopted to assess both the measurement and structural models (Anderson and Gerbing, 1988). The first step involved a comprehensive evaluation of the model’s reliability and validity. Subsequently, fit coefficients and path coefficients of the hypothesized model were measured, and the existence of the mediating effects was examined.

The researcher checked for Common Method Variation (CMV) by comparing the chi-square values and degrees of freedom differences between Model 1 and Model 2, following the suggestion of Mossholder et al. (1998). The results showed that the chi-square value for Model 1 was 2871.1, with 119 degrees of freedom, and a *p*-value less than 0.001. For Model 2, the chi-square value was 187.5, with 113 degrees of freedom, and a p-value less than 0.001. The ratio of the chi-square difference to the degrees of freedom difference between the two models was 447.3, indicating that the fit of Model 1 was proportional to Model 2. Therefore, it can be concluded that there is no evidence of univariate structure, suggesting that CMV does not exist in this study.

## 4 Results

### 4.1 Assessment of the measurement model reliability and validity

This study examined reliability and discriminant validity by calculating the Composite Alpha (Cα) and Composite Reliability (CR) coefficients for latent variables (Fornell and Larcker, 1981). As presented in Table 2, the Cα coefficients for each variable range between 0.861 and 0.964, with CR values exceeding 0.8 on average, and the Average Variance Extracted (AVE) for each variable falls between 0.624 and 0.844. Therefore, all variables demonstrate high reliability and convergent validity. Additionally, as indicated in Table 3, the correlation coefficients for all variables are lower than the square root of AVE, suggesting robust discriminant validity among the variables.

**Table 2:** Reliability and validity test.

Items	Loadings	Cα	AVE	CR
Physical activity (PA)		0.861	0.684	0.866
PA1	0.844			
PA2	0.846			
PA3	0.745			
Emotional intelligence (EI)		0.963	0.825	0.949
EI1	0.954			
EI2	0.877			
EI3	0.920			
EI4	0.878			
EI5	0.947			
Interpersonal forgiveness (IF)		0.866	0.624	0.868
IF1	0.883			
IF2	0.779			
IF3	0.748			
IF4	0.734			
Positive emotion (PE)		0.964	0.844	0.964
PE1	0.936			
PE2	0.923			
PE3	0.897			
PE4	0.931			
PE5	0.905			

Cα, Cronbach’s alpha; AVE, average variance extracted; CR, composite reliability.

**Table 3:** Pearson correlations and square roots of AVE for discriminant validity test.

Construct	Physical activity (1)	Emotional intelligence (2)	Interpersonal forgiveness (3)	Positive emotion (4)
1	**(0.827)**			
2	0.523^**^	**(0.908)**		
3	0.544^**^	0.637^**^	**(0.790)**	
4	0.625^**^	0.641^**^	0.574^**^	**(0.919)**

The square root of the average variance extracted (AVE) is in the diagonals (bold); off diagonals is a Pearson’s correlation of contracts. ^**^p < 0.01.

### 4.2 Hypothesis testing results

**Figure 2:**
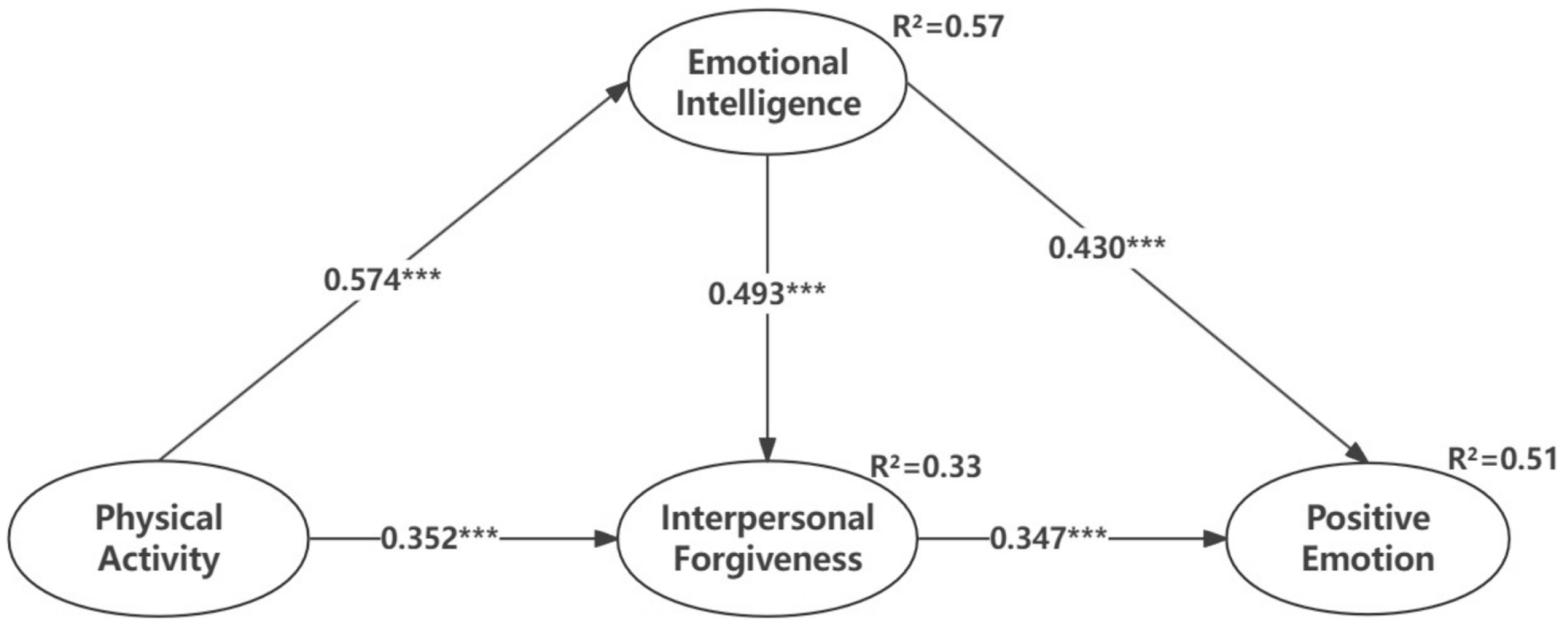
Structural path model. ^***^p < 0.001. Standardized coefficients are reported.

Firstly, the structural equation model’s error and residual terms did not exhibit negative values, indicating that the model did not violate estimation principles. Secondly, both the data and the structural equation model demonstrated high goodness of fit (*χ*^2^/df = 2.233, GFI = 0.938, AGFI = 0.917, NFI = 0.969, RMSEA = 0.051), significantly surpassing recommended thresholds. Thirdly, according to the Pearson correlation results in Table 3, there were significant correlations among the independent, mediating, and dependent variables, supporting the validation of the hypotheses. Fourthly, the structural path model in Figure 2 illustrated that the relationships between physical activity and emotional intelligence (*β* = 0.574, *p* < 0.001, supporting H1), physical activity and interpersonal forgiveness (*β* = 0.352, *p* < 0.001, supporting H2), emotional intelligence and interpersonal forgiveness (*β* = 0.493, *p* < 0.001, supporting H3), emotional intelligence and positive emotion (*β* = 0.430, *p* < 0.001, supporting H4), and interpersonal forgiveness and positive emotion (*β* = 0.347, *p* < 0.001, supporting H5) were all statistically significant.

Researchers hypothesized that physical activity affects positive emotion through both emotional intelligence and interpersonal forgiveness, and the study utilized bootstrapping to test the mediating effects (Bollen and Stine, 1990). The standardized results of the 5,000-bootstrap samples with a 95% confidence interval are presented in Table 4: the absolute values of the *Z*-statistics for the PA → PE mediating effect exceeded 1.96, indicating no zero values within the 95% confidence interval. Additionally, emotional intelligence and interpersonal forgiveness significantly influenced the relationship between physical activity and positive emotion (standardized indirect effect = 0.467, *p* < 0.001), supporting H6. The findings suggest that students with higher levels of physical activity, emotional intelligence, and interpersonal forgiveness also exhibit higher levels of positive emotion.

**Table 4:** Indirect effects.

	Point estimate	Product of coefficients		Bootstrapping		
				Bias-corrected 95% CI		Two-tailed significance
		*SE*	*Z*	Lower	Upper	
PA → EI → IF→PE	0.467	0.045	10.378	0.378	0.554	<0.001
PA → EI → PE	0.171	0.041	4.171	0.205	0.387	<0.001
PA → IF→PE	0.283	0.046	6.152	0.100	0.262	<0.001

Standardized estimations of 5,000 bootstrap samples.

## 5 Discussion

### 5.1 Theoretical contribution

This study makes several contributions to the theory of adolescent mental health. Firstly, existing research predominantly focuses on the impact of physical activity on adolescents’ anxiety (Reddy et al., 2018), depression (Young et al., 2019; Tran et al., 2023), sleep quality (Shen et al., 2018; Albrecht et al., 2022) and physical health (Godoy-Cumillaf et al., 2023). The direct exploration of positive emotions in adolescents is relatively scarce. By investigating the influence of physical activity on adolescents’ emotional intelligence, interpersonal forgiveness, and positive emotions, this study addresses a critical research gap. It provides a solid theoretical foundation for the relationship between physical activity and interpersonal forgiveness, offering a more targeted perspective and enriching the theoretical research in positive psychology. The study posits that positive emotions in adolescents can promote physical activity, and in turn, positive physical activity motivations can enhance their positive emotions—a mutually reinforcing process conducive to the holistic development of adolescents’ mental and physical well-being.

Moreover, this research takes a step further by primarily intervening in physical health courses in school sports to enhance adolescents’ positive emotions. It offers a theoretical basis for the practical implementation of physical activity interventions among adolescents. Adolescents, facing academic pressure, parental expectations, competition, and complex interpersonal relationships during puberty, often have limited time for physical activity. The reduction in physical activity makes it challenging for them to achieve the full benefits for health promotion (Sallis, 2000). Therefore, they require more theoretical guidance for practice. The study reveals that participation in physical activity has a profound impact on adolescents’ positive emotions. Engaging in sports not only improves their emotional management skills but also enhances their interpersonal forgiveness abilities, contributing to the establishment of positive interpersonal relationships.

Secondly, the results of this study establish a solid theoretical foundation for the positive effects of physical activity on adolescents’ emotional intelligence, interpersonal forgiveness, and positive emotions. It is noteworthy that physical activity has the greatest impact on emotional intelligence, followed by interpersonal forgiveness. This observation can be attributed to the mediating roles that emotional intelligence and interpersonal forgiveness play in the relationship between physical activity and positive emotions. While physical activity directly influences adolescents’ positive emotions, it also indirectly affects their positive emotions by influencing levels of emotional intelligence and interpersonal forgiveness. Figure 2 visually illustrates the relationships between these variables, supporting the findings of Chan et al. (2019) and Ubago-Jiménez et al. (2019), revealing that emotional intelligence and interpersonal forgiveness jointly explain 51% of the variance in positive emotions. This study provides a promising pathway for researching the relationship between physical activity and positive emotions.

### 5.2 Practical implications

Given the significant impact of physical activity on adolescents’ emotional and interpersonal well-being as identified in our study, it is critical that concerted efforts are made across various societal domains to promote physical activities among this demographic. These efforts should be multifaceted, addressing not only the availability of opportunities for physical activity but also the quality and inclusivity of these opportunities.

Firstly, government authorities should recognize the pivotal role of physical activity in enhancing the overall health of adolescents. Elevating the status of physical education in school curricula, increasing the weightage of sports in exams, and incorporating physical education into high school education are essential steps. Encouraging policymakers to integrate positive practices into school curricula as effective and easily implementable tools for enhancing adolescent mental health is imperative. Government departments should actively enact policies, urging and incentivizing adolescents to actively engage in physical activities, enhancing their physical fitness. Additionally, enhancing regulation of physical education in schools ensures effective implementation of policies rather than merely fulfilling formalities. Financial and logistical support from the government to schools is vital to ensure adequate resources for the development of sports programs. Furthermore, public awareness campaigns should be initiated to promote the benefits of sports activities and elevate public awareness of sports culture.

Secondly, schools should actively implement government regulations and policies, translating the emphasized issues into tangible actions. Schools need to shift from a purely academic evaluation system to prioritize holistic education. Many schools in China tend to prioritize students’ academic performance, neglecting their physical health development, leading to the phenomenon of physical education classes being frequently replaced by other subjects. This significantly reduces adolescents’ physical activity time, impacting their overall development. Focusing on the development of school sports, increasing the proportion of physical education courses, and providing sufficient time for physical activity during school hours are essential. Schools should also enhance the training of physical education teachers and improve the quality of physical education courses. Diversifying the content of physical education courses, adapting teaching methods to individual students, and encouraging students to collaborate, communicate, and actively participate in sports activities are crucial. To address the issue of inadequate facilities and equipment, schools should increase investment in sports culture construction, ensure the allocation and management of basic facilities such as sports venues and equipment, creating a conducive environment for students’ physical education and extracurricular activities.

Thirdly, family awareness of the concept of sports plays a crucial role in adolescents’ participation in physical activities. The family is a key factor in interventions aimed at enhancing adolescents’ self-awareness capabilities. Parents’ self-awareness is closely related to the extent of children’s participation in sports activities. Therefore, parents should establish correct educational values, actively collaborate with schools in implementing physical education programs, and encourage adolescents to participate in diverse extracurricular sports activities. Moreover, parents should strive to create a positive sports atmosphere at home, actively supporting and encouraging adolescents to engage in physical activities, participating in sports together, fostering healthy sports habits, and instilling awareness of lifelong physical fitness.

### 5.3 Limitations

However, despite the positive contributions of this study, it is not without limitations. Firstly, it is a cross-sectional study, preventing us from drawing conclusions about causation. Therefore, longitudinal studies should delve deeper into examining these relationships. Additionally, we recommend that future research employs longitudinal designs to validate the findings of our cross-sectional study. Secondly, the sample used and its size also limit the generalizability of the results. Furthermore, this study primarily focused on a sample of Chinese adolescent students, highlighting the need for an understanding of cultural differences when exploring similar topics. Thirdly, this study did not ask respondents to record the number of days they engage in physical activities, which may affect the accuracy of our assessment of the relationship between exercise frequency, intensity, and positive emotions. Future research should consider collecting more detailed data on physical activity, including duration, frequency, and intensity, to more accurately analyze the impact of physical activity on psychological health. Fourthly, to simplify the questionnaire and reduce respondent fatigue, several items with similar expressions were removed from the scale. Although this decision was based on the redundancy among these items, future research should determine whether to delete or retain items. This could include more comprehensive pilot testing or statistical analysis to ensure that such modifications do not compromise the reliability or validity of the scale. We also recommend that subsequent studies explore the impact of these changes across different cultural contexts to validate the universality and applicability of the scale. Additionally, incorporating these detailed dimensions could further reveal the specific effects of physical activity on emotional intelligence and interpersonal forgiveness, providing a theoretical basis for developing more effective intervention measures.

## 6 Conclusion

Addressing the research objectives, the results of this study underscore the significant impact of physical activity on the emotional intelligence, interpersonal forgiveness, and positive emotions of adolescents. Therefore, it is crucial for governmental bodies, schools, and families to collaboratively foster the comprehensive development of the physical and mental well-being of adolescents.

In conclusion, the results of this study confirm the positive impact of physical activity on the emotional intelligence of adolescents, as well as its positive influence on interpersonal forgiveness, laying the theoretical foundation for future research on the relationship between physical activity and interpersonal forgiveness. Moreover, physical activity has been proven to have a positive effect on the positive emotions of adolescents. Through collaborative efforts from the government, schools, and families, a conducive environment and conditions can be created to promote the physical and mental health growth of adolescents.

## Data availability statement

The raw data supporting the conclusions of this article will be made available by the authors, without undue reservation.

## Ethics statement

The studies involving humans were approved by Ethics Committee of the School of Physical Education of Hunan University of Science and Technology (No. ECBPEHNUST 2023/0011). The studies were conducted in accordance with the local legislation and institutional requirements. Written informed consent for participation in this study was provided by the participants’ legal guardians/next of kin.

## Author contributions

SY: Conceptualization, Investigation, Methodology, Writing – original draft, Writing – review & editing. LJ: Investigation, Resources, Writing – original draft, Writing – review & editing. QH: Resources, Supervision, Writing – original draft, Writing – review & editing. HW: Conceptualization, Funding acquisition, Project administration, Writing – original draft, Writing – review & editing.
